# *Ciona intestinalis* as a Marine Model System to Study Some Key Developmental Genes Targeted by the Diatom-Derived Aldehyde Decadienal

**DOI:** 10.3390/md13031451

**Published:** 2015-03-17

**Authors:** Anna Lettieri, Rosaria Esposito, Adrianna Ianora, Antonietta Spagnuolo

**Affiliations:** Stazione Zoologica Anton Dohrn, Villa Comunale, 80121 NAPOLI, Italy; E-Mails: anna.lettieri@szn.it (A.L.); rosaria.esposito@szn.it (R.E.)

**Keywords:** ascidian, Hox, ParaHox, stress, glutathione (GSH), development

## Abstract

The anti-proliferative effects of diatoms, described for the first time in copepods, have also been demonstrated in benthic invertebrates such as polychaetes, sea urchins and tunicates. In these organisms PUAs (polyunsaturated aldehydes) induce the disruption of gametogenesis, gamete functionality, fertilization, embryonic mitosis, and larval fitness and competence. These inhibitory effects are due to the PUAs, produced by diatoms in response to physical damage as occurs during copepod grazing. The cell targets of these compounds remain largely unknown. Here we identify some of the genes targeted by the diatom PUA 2-*trans*-4-*trans*-decadienal (DD) using the tunicate *Ciona intestinalis*. The tools, techniques and genomic resources available for *Ciona*, as well as the suitability of *Ciona* embryos for medium-to high-throughput strategies, are key to their employment as model organisms in different fields, including the investigation of toxic agents that could interfere with developmental processes. We demonstrate that DD can induce developmental aberrations in *Ciona* larvae in a dose-dependent manner. Moreover, through a preliminary analysis, DD is shown to affect the expression level of genes involved in stress response and developmental processes.

## 1. Introduction

Diatoms are a major class of unicellular algae that have traditionally been considered essential in sustaining the marine food chain. This paradigm was challenged over a decade ago with the discovery that they produce polyunsaturated aldehydes (PUAs) that induce abortions and teratogenesis in grazing predators [1]. PUAs and, in particular, 2-*trans*-4-*trans*-decadienal (DD) have been shown to decrease hatching success and to induce strong developmental aberrations in different marine organisms. The aberrant phenotypes observed range from malformed or reduced number of feeding or swimming appendages in copepods [2], shortening of the apical spicules and arms [3] and stunted and asymmetrical larval arms [4] in sea urchins, incomplete ciliary band formation in polychaete larvae [4] and stunted elongation of the tail in ascidians [5]. The degree and frequency of asymmetrical (teratogenic) development increases with increasing PUA concentrations, and there is a clear stage-specific effect, with earlier larval stages being the most affected [6]. However, despite the large number of studies showing that diatom oxylipins negatively impact invertebrate reproduction and development, information on the molecular mechanisms and the associated genes targeted by this PUA during development of marine organisms is lacking.

In the present study, we use the tunicate *Ciona intestinalis* for a detailed analysis of the effect of DD on embryonic development. Having been used for more than a century as a model for embryological studies, more recently *Ciona* has become a key organism for evolutionary and developmental biology studies thanks to a number of computational tools, techniques and genomic resources available for this species. The annotated genome is indeed relatively small (about 160 Mbp), consisting of 14 pairs of chromosomes with about 16,000 genes. Embryogenesis in *C. intestinalis* is comparatively simple and well documented, characterized by a stereotyped development that is based on invariant early cell lineages and a remarkably small number of cells. Moreover, a very large number of synchronously developing embryos can be obtained, which are anatomically simple, easy to manipulate and analyze for morphological aberrations. All these characteristics are thus instrumental for the adoption of *Ciona* as a “model system” for toxicological studies on marine organisms.

Here we conducted a detailed study on the effects of DD on *Ciona* embryo development. Furthermore, using a qPCR approach, we compared the expression levels, between control and DD treated embryos, of some selected representatives of two classes of transcripts: genes involved in stress response and genes involved in development. For the first class, the role played by the glutathione system in protecting cells from oxidative stress and contributing to a favorable redox environment is well known [7]. Since genomic analyses have revealed a conserved glutathione (GSH) homeostasis pathway in *C. intestinalis* [8], we analyzed the changes in the expression levels of two genes coding for two enzymes acting at two different levels of the GSH pathway, namely GCLM (subunit of the γ-glutamyl-cysteine ligase) and GST (Glutathione *S*-transferase) [7]. For the second class, we focused our attention on representatives of Hox/ParaHox factors, a wide family of genes that are fundamental for development and patterning throughout animal phylogeny [9]. In particular we analyzed changes in the expression levels of *hox1* and *gsx*, as representatives of anterior, and *hox12* and *cdx*, as representatives of posterior Hox and ParaHox classes, respectively. The ultimate goal of our study was to better understand the toxic effects of DD on *Ciona* development and to use this information to explore the intricate world of the genes that are potentially affected by DD in marine organisms.

## 2. Results

**Figure 1:**
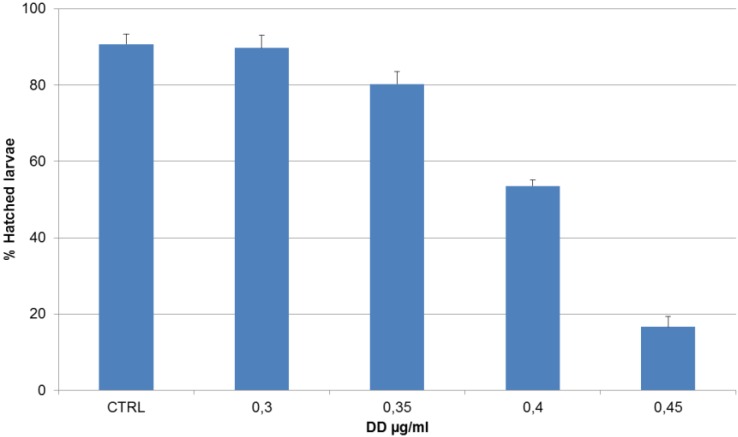
Percentage of hatched larvae *vs.* increasing 2-*trans*-4-*trans*-decadienal (DD) concentrations after treatment on newly fertilized *Ciona* eggs. Data are expressed as mean percentage of the number of hatched larvae ± standard deviation (sd). A value of *p* < 0.05 was considered statistically significant in a one-way ANOVA test used to compare the treatments with the control.

Treatment of virgin oocytes with increasing DD concentrations resulted in a short delay in development, which was not quantified. Pretreatment of eggs prior to fertilization had no effect on hatching success and on larval phenotype. Also in the case of DD treatment of the sperm before fertilization, no effect was observed on hatching success and larval phenotype. When newly fertilized eggs were incubated with DD concentrations ranging from 0.30 to 0.50 µg·mL^−1^, a decreasing number of hatched larvae were observed (Figure 1).

**Figure 2:**
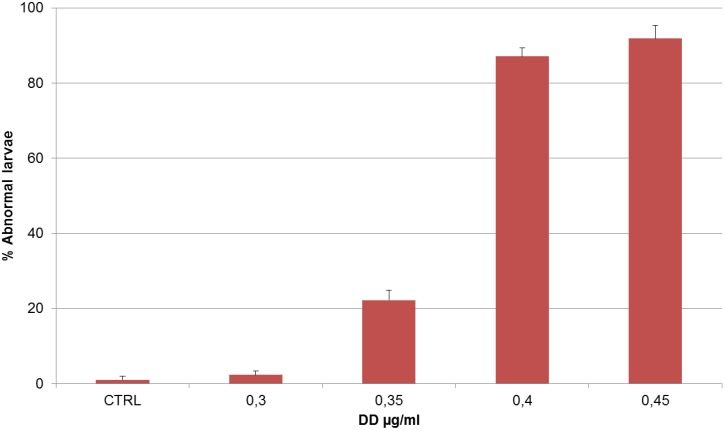
Percentage of abnormal larvae *vs.* increasing DD concentrations, after treatment on newly fertilized *Ciona* eggs. Data are expressed as mean percentage of number of abnormal larvae ± standard deviation (sd). A value of *p* < 0.05 was considered statistically significant in a one-way ANOVA test used to compare the treatments with the control.

This effect was dose-dependent. Moreover, at these DD concentrations, a higher number of abnormal hatched larvae were observed (Figure 2). In particular, at the lowest DD concentration tested (0.3 µg·mL^−1^) larvae had a normal phenotype comparable to the control; at higher DD concentrations (ranging from 0.35 µg·mL^−1^ to 0.40 µg·mL^−1^), the phenotype became abnormal.

**Figure 3:**
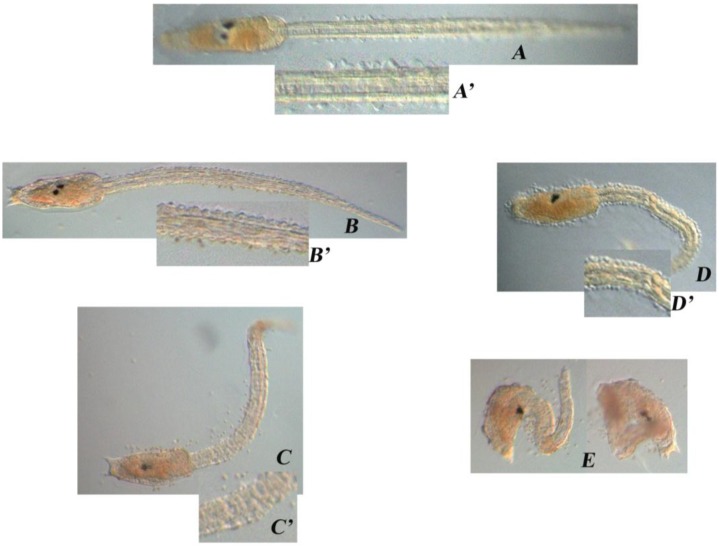
Aberrant *Ciona* phenotypes. Hatched larvae after DD treatment on fertilized *Ciona* eggs ((**A**) CTRL; (**B**) 0.30 µg·mL^−1^; (**C**) 0.35 µg·mL^−1^; (**D**) 0.40 µg·mL^−1^; (**E**) 0.45 µg·mL^−1^). (**A’**–**D’**) Magnification of the tails showing the progressively altered structure (**B’**–**D’**) compared to the control (**A’**).

Aberrations occurred mainly on the larval tail (Figure 3). In *Ciona*, the combination of epidermal, muscle, notochord and caudal neural tube cells is sufficient for the elongation of the tail bud. In controls, the tail shows its typical structure made of vacuolated notochord cells, muscle cells running the length of the tail on either side of the notochord, the ventral endodermal strand, the dorsal neural tube and the surrounding epidermis (Figure 3A). In DD treated larvae (0.35 µg·mL^−1^) the overall tail organization appeared to be affected and the phenotype became more severe at higher DD concentrations (0.40 µg·mL^−1^). In these embryos, indeed, the tail was even shorter, kinked and disorganized, while the trunk seemed quite normal (Figure 3C,D). At DD concentrations of 0.45 µg·mL^−1^, larvae were completely disorganized and failed to hatch (Figure 3E). At the highest concentration tested (0.50 µg·mL^−1^) no development was observed. The larval phenotypes are described in the Table 1. Malformed hatched larvae showed an abnormal swimming behavior; they could move their tail but could not swim forward, as they would normally do, but could only trace circles around themselves.

**Table 1:** Description of the aberrations in *Ciona* larvae. Left column reports DD concentrations in µg·mL^−1^; right column reports aberrations after DD treatment on newly fertilized eggs (capital letters refer to the corresponding picture in Figure 3).

DD (µg/mL)	Treatment on Fertilized Eggs
–	Normal phenotype (A)
0.30	Normal phenotype (B)
0.35	Tail was shorter than the control, kinked and disorganized (C)
0.40	Tail was even shorter, kinked and disorganized; the trunk seemed quite normal (D)
0.45	Larvae were completely disorganized and failed to hatch (E)
0.50	No development

DD treatment also induced a developmental delay. This delay was visible throughout the stages of development and was dose-dependent. In fact, at 24 hpf (hours post fertilization) there was more than 90% hatching in control embryos and in those treated with 0.30 µg·mL^−1^, while in those treated with 0.35, 0.40 and 0.45 µg·mL^−1^ DD, the percentage of the developed embryos that completed the hatching was only 20%, 10% and 1%, respectively. It took another 30 min (for 0.35 µg·mL^−1^ DD), 1 h (for 0.40 µg·mL^−1^ DD) and 1.5 h (for 0.45 µg·mL^−1^ DD) for the developed embryos to complete the hatching. At 0.50 µg·mL^−1^, none of the larvae hatched and development was blocked. To understand if the effects of DD were reversible, two wash steps were introduced after treatment. Rinsing 8-cell stage embryos twice for five minutes did not reverse the effect of DD at any of the DD concentrations tested.

### 2.1. Stress Genes

**Figure 4:**
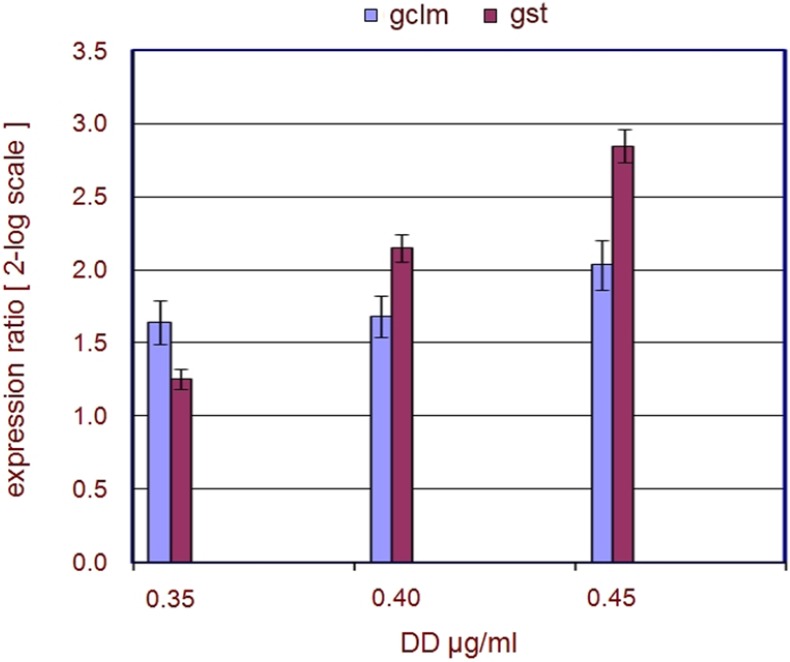
Regulation of stress genes. The plot represents the regulation of stress genes after DD treatment on fertilized *C. intestinalis* eggs. The expression ratios (from the Pfaffl equation) [13] ± standard deviation (sd) are indicated on the y-axis. DD concentrations are reported on the x-axis and are given as µg·mL^−1^. The legend on the top indicates the colors associated to the genes.

Glutathione (GSH) associated metabolism is central in detoxification and is probably the most important cellular defense against Reactive Oxygen Species (ROS) and their toxic products, thus allowing the living organisms to prevent and defend itself against any source of insult [10]. GSH is synthesized from two consecutive ATP-dependent reactions [11]. In the first step γ-glutamylcysteine (γ-EC) is formed from l-glutamate and l-cysteine by the Glutamate Cysteine Ligase (GCL), a dimeric holoenzyme composed of a catalytic (GCLC) and a modifier (GCLM) subunit. The second step is catalyzed by glutathione synthetase, which adds glycine to the *C*-terminal of γ-EC forming GSH. Glutathione exists in the thiol-reduced (GSH) and disulfide-oxidized (GSSG) forms [12]. In the GSH cycle, most of the detoxication reactions are mediated by Glutathione *S*-transferases (GSTs) enzymes that catalyze the conjugation of toxic xenobiotics and oxidatively produce compounds to reduced glutathione, which facilitates their metabolism, sequestration, or removal [7]. The qPCR experiments included the analysis of expression intensity of transcripts coding for two enzymes fundamental for the GSH cycle, namely GCLM, one of the subunits of the GCL enzyme, and GST. qPCR data demonstrated that embryos (at the late tailbud stage-9 hpf) after DD treatment showed an increased expression level of the genes involved in the stress response (Figure 4). In particular, *gclm* was already significantly (≥1.5) up-regulated at the lowest DD concentration tested (0.35 µg·mL^−1^); up-regulation increased as DD concentration increased, while *gst* was significantly (≥1.5) up-regulated at DD concentrations ≥0.35 µg·mL^−1^. The qPCR data on stress genes are strongly supported by a differential expression analysis (Table 2), conducted by microarray experiments, currently in progress in laboratory (personal communication, data not published yet).

**Table 2:** Microarray results. Table showing preliminary results of a microarray analysis. For each gene the Fold Change value (FC) and the *p*-value (*p*) are reported at two different DD concentrations (0.40 and 0.45 µg·mL^−1^).

Genes	FC 0.40	FC 0.45	*p* 0.40	*p* 0.45
***gclm***	4.6	5.1	0.003308	0.049152
***gst***	5.2	8.4	0.040746	0.038839

### 2.2. Developmental Genes

**Figure 5:**
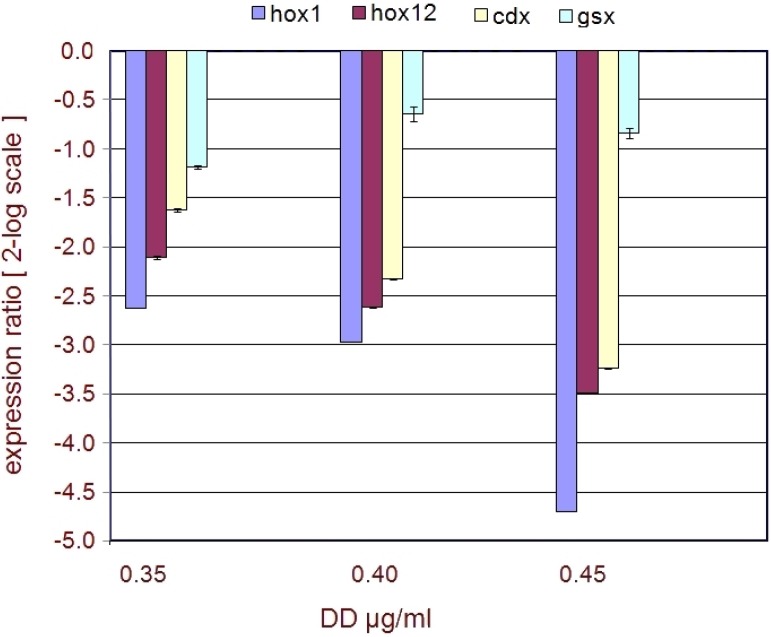
Regulation of developmental genes. The plot represents the regulation of developmental genes after DD treatment on fertilized *C. intestinalis* eggs. Expression ratios (from the Pfaffl equation) ± standard deviation (sd) are indicated on the y-axis. DD concentrations are reported on the x-axis and are given as µg·mL^−1^. The legend on the top indicates the colors associated to the genes.

Hox and ParaHox genes, a subfamily of the homeobox family of transcription factors, are major regulators of animal development. They usually show a clustered genomic organization and specify developmental fates along the anterior-posterior body axis of all animals in which they have been examined [9]. *Ciona* genome contains representatives of nine classes of Hox family genes [14], and the three representatives of ParaHox subfamily, *gsx*, *xlox* and *cdx* [15]. For qPCR experiments we focused our attention on the expression intensity of *hox1* and *hox12*, as representatives of *Ciona* anterior and posterior Hox class genes, respectively [14], and of *gsx* and *cdx*, as representatives of *Ciona* anterior and posterior ParaHox class genes, respectively. qPCR data indicated that, unlike the stress related genes, the expression levels of *hox1*, *hox12* and *cdx* decreased after DD treatment, while the profile of *gsx* did not seem to be particularly affected (Figure 5). *hox1*, *hox12* and *cdx* genes were significantly (≤−1.5) down-regulated already in the presence of 0.35 µg·mL^−1^ DD. Moreover they were further down-regulated at higher DD concentrations. The qPCR data on developmental genes are strongly supported by a differential expression analysis (Table 3), conducted by microarray experiments, currently in progress in laboratory (personal communication, data not published yet).

**Table 3:** Microarray results. Table showing preliminary results of a microarray analysis. For each gene the Fold Change value (FC) and the *p*-value (*p*) are reported at two different DD concentrations (0.40 and 0.45 µg·mL^−1^).

Genes	FC 0.40	FC 0.45	*p* 0.40	*p* 0.45
*hox1*	−2.7	−5.3	0.025202	0.001851
*hox12*	−1.7	−2.6	0.03697	0.007098
*cdx*	−1.6	−2.4	0.039887	0.00173
*gsx*	-	−1.1	-	0.20408

## 3. Discussion

Recent studies have suggested that DD has the potential to induce strong developmental aberrations in different marine organisms. In this study we used *C. intestinalis* as a key marine model to evaluate the phenotypic effects of DD during embryogenesis up to the larval stage and explore possible toxic mechanisms. Tunicates such as *C. intestinalis* may come into contact with diatom DD or other PUAs in the field at the end of a bloom, with the mass sinking of diatoms to the sediment. As a strong filter feeder that grazes mainly on phytoplankton cells, including diatoms, *Ciona* may accumulate PUAs through feeding or be exposed to local concentrations of these compounds that may affect growth performance. This is of considerable ecological relevance considering the importance of diatom blooms in nutrient-rich aquatic environments.

During our tests, we used DD concentrations ranging 0.2–2.0 µg·mL^−1^ that are much higher than those measured in the field [16]. However, it is worth mentioning that in nature the animals are exposed to a longer chronic (even if lower) exposure and this allow them to accumulate the compound. Thus, to mimic the same “natural” situation in the tight time of a laboratory experiment, it is often necessary to increase the concentrations of the drug of interest. Notably, PUA concentrations we used fall in the range that have been tested on different organisms in other laboratory experiments [17].

The results demonstrate that DD exposure causes dose dependent embryonic lethality and larval malformations mostly at the level of tail development and organization. In addition, by qPCR experiments, we assessed that in *Ciona* GSH metabolism is undoubtedly one of the mechanisms that is immediately activated in response to DD. More importantly, qPCR data indicated that DD exposure alters the expression of fundamental embryogenesis-related factors, namely representatives of Hox and ParaHox gene families, thus offering new clues to analyze the potential impact of toxic compounds in the wildlife.

### 3.1. Embryonic Toxicity

When virgin oocytes were treated with increasing DD concentrations (0.20, 0.40, 0.60 µg·mL^−1^) and were then fertilized with untreated sperm, a small decrease in percentage hatching was observed, but hatched larvae appeared normal. These results were different from the results previously described on *Ciona* by Tosti [5]. These authors observed a decrease in the first cleavage after treatment of oocytes with increasing DD concentrations ranging from 0.10 to 1.5 µg·mL^−1^. Moreover, they observed an increase in the percentage of abnormal larvae at DD concentrations between 0.10 and 0.20 µg·mL^−1^. The difference between the two results (Tosti and our results) may be due to differences in the treatments. In fact, in the case of experiments conducted by Tosti and co-workers, eggs were dechorionated before treatment, whereas in the present study eggs were not dechorionated. One possibility may be that the chorion acts as a physical barrier (up to certain points) to external environmental vagaries (including toxic substances such as DD) or that protective strategies (*i.e.*, membrane transporters), are activated in *Ciona* embryos, as demonstrated in other animal embryos [18].

The aberrant phenotype induced by DD treatment was not reversible at any of the concentrations tested, even the lowest (0.30 µg·mL^−1^). These results differ from those obtained by Tosti *et al.* [5] who demonstrated that the effect of 1 µg·mL^−1^ DD on development was completely reversible if the oocytes were rinsed 2 min after fertilization. Rinsing fertilized oocytes after 10 min did not reverse the effect of DD on development. Here too, the difference between the two experiments could be due to differences in the two treatments. In Tosti *et al.* [5], virgin oocytes were treated with DD, whereas in our study DD treatments were performed after fertilization. This could imply that the processes that occur after fertilization are affected by DD in a more severe manner.

One of the most interesting findings in our study was a developmental dose-dependent delay after DD treatment of eggs soon after fertilization. The same delay has also been observed in sea urchins [3]; in these experiments DD retarded development at concentrations lower than 1.32–5.26 μM (0.20–0.80 µg·mL^−1^, respectively), whereas cleavage was blocked at concentrations of 6.58 μM (1 µg·mL^−1^).

We also found that hatching success decreased after DD treatment in a dose-dependent manner. This effect has also been observed in other marine organisms. For example, in the sea urchin *Paracentrotus lividus* DD exerts a very strong dose-dependent effect on hatching success. At concentrations of about 3.0 μM (0.50 µg·mL^−1^), DD reduced hatching viability to <50%; total inhibition of hatching viability occurred at DD concentrations of 3.95 μM (0.60 µg·mL^−1^) [3].

At the phenotypical level, aberrations in the development of *Ciona* seemed localized mostly in the tail, while the trunk was apparently normal. Indeed, increased concentrations of DD (0.35–0.45 µg·mL^−1^), resulted in larvae with progressively shorter tails, showing an overall disorganized structure, that was compromised to the point that larvae could no longer swim correctly; in fact, they could move their tail but they could not swim forward but only in circles. This abnormal swimming behavior has also been observed in copepods [19]. Some authors found that nauplii hatched from diatom-fed females were generally deformed and expressed marked morphological asymmetry [5]. Most deformed nauplii died after hatching, whereas those that survived displayed greatly impaired swimming behavior [20]; other authors [21] recorded that about 80% of spawned eggs of the copepod *Calanus simillimus* underwent abnormal development resulting in either abortions or production of asymmetrical larvae. The severity of swimming behavior was directly linked to an abnormal development with asymmetrical nauplii that tended to turn in the direction opposite to the most developed swimming appendages [19]. Such a change in locomotory capacity impaired feeding efficiency and escape behavior [22].

A further interesting finding was that after DD treatment *Ciona* larvae were normal at a concentration of 0.30 µg·mL^−1^, whereas an abnormal phenotype was observed immediately beyond this concentration, at 0.35 µg·mL^−1^. In other words, there was an abrupt passage in the appearance of abnormal phenotypes indicating a DD threshold concentration at which this phenotype appears, thus suggesting that endogenous defense mechanisms, activated in response to the DD, are able to protect *Ciona* embryos up to the threshold level of 0.30 µg·mL^−1^.

### 3.2. Stress Related Genes

qPCR results demonstrate that genes involved in the stress response (gclm-gst) were up-regulated after treatment with DD concentrations (0.35, 0.40, 0.45 µg·mL^−1^). In particular, gclm was up-regulated already at 0.35 µg·mL^−1^ DD; embryos may therefore try to immediately contrast the stress induced by DD by alerting the defense mechanisms that, initially, appear to be effective in counteracting DD. This is supported by the relatively low numbers (<50%) of abnormal larvae detected after treatment with 0.35 µg·mL^−1^. At higher DD concentrations the defense mechanisms are inactivated, as revealed by the high up-regulation of both *gclm* and *gst*. Both genes are no longer able to protect *Ciona* embryos, which appear completely disorganized and fail to hatch at 0.45 µg·mL^−1^ DD concentrations. Collectively these data indicate that GSH metabolism is undoubtedly one of the mechanisms that is immediately activated in *Ciona* embryos in response to DD exposure, further confirming the role of GSH as the most versatile nucleophile to contrast a plethora of chemical challenges encountered by all life forms.

### 3.3. Developmental Genes

The overall phenotype exhibited by *Ciona* larvae indicates that DD treatment could somehow impair the function of developmentally relevant genes involved in the organization of body axis structures, along the anterior posterior axis and, particularly, at the level of tail formation. In this regard, Hox and ParaHox factors, being the major regulators of animal development, represent one of the “first choice gene families” to be considered, in the light of exploring the possible molecular mechanisms responsible for the adverse effects exerted by DD. qPCR experiments clearly indicated that a down-regulation of *hox1*, *hox12* and *cdx* was induced by DD treatment of newly fertilized eggs already at 0.35 µg·mL^−1^ DD, while the levels of expression of *gsx* appeared almost unaltered. As previously pointed out, *hox12* and *cdx* belong to the posterior class of Hox and ParaHox gene families, respectively. *Hox12* is expressed, at the tailbud stage, in the end of the posterior nerve cord and epidermis [23] and antisense morpholino injection experiments indicated that this gene plays an important role in tail development, by controlling the elongation of both, the tail nerve cord and the tail itself, and the morphology of the tail end and of the epidermal cells at the tail tip [24]. *Cdx* is also expressed, at the tailbud stage, in the tail epidermis, endodermal strand and tail nerve cord, and expression of a dominant negative *cdx* causes defects in neural tube formation and tail elongation [25]. It is intriguing to note that the interference with *hox12* and *cdx* transcripts results in a phenotype that mirrors the DD phenotype, thus reinforcing the qPCR data and suggesting an involvement of these factors in the phenotypic effects of DD during *Ciona* embryogenesis.

Concerning the anterior representative genes, hox1 shows a strong down-regulation already at 0.35 µg·mL^−1^ DD, while gsx does not seem to be strongly affected. It is important to note that in *Ciona* *gsx*, the most anterior ParaHox gene is expressed from the early stages of development and up to the tailbud stage, in the trunk of embryos, at the level of the sensory vesicle [26]. *Hox1*, the most anterior Hox gene, is instead not expressed in the trunk but in the anterior part of the tail, and is localized in the visceral ganglion, extending to the posterior along the nerve cord, and in the epidermis surrounding the neck region in the tailbud embryos [23]. Thus, the altered qPCR profile of *hox1*, with respect to that of *gsx*, appears to fit with the expression territories of these genes (*hox1* in the tail and *gsx* in the trunk) and in turn with the phenotypic effects of DD exerted mostly at the level of the tail.

## 4. Experimental Section

### 4.1. Collection of Animals and Harvesting of Gametes

*Ciona intestinalis* were collected from the Gulf of Naples (Italy), from January to May, and maintained in tanks with constantly flowing seawater for seven days under constant light to accumulate gametes. Eggs and sperm were obtained surgically from the gonoducts. Eggs were fertilized by mixing with sperm from other individuals for 15 min in filtered seawater at room temperatures, in 1.5 mL Eppendorf conical tubes.

### 4.2. Decadienal Solutions

2-*trans*-4-*trans*-Decadienal (DD) was purchased from Acros Organics (part of Thermo Fischer Scientific, Geel, Belgium). A stock solution of 6 mg·mL^−1^ was prepared by dissolving 4.1 µL DD in 500 µL DMSO. Work solution of 0.6 mg·mL^−1^ was obtained by diluting appropriate volumes of stock solution in DMSO. DMSO had no effect on *C. intestinalis* up to 1% DMSO in filtered seawater. Treatment solutions were prepared by adding appropriate volumes of working solution to filtered seawater.

### 4.3. Exposure to DD Prior to Fertilization

Eggs and sperm were treated with DD before fertilization in two different experiments. In the first experiment, approximately five hundred eggs (500 eggs for each DD concentration tested and 500 for the control) were collected from the gonoduct and were placed into 60 mm cell culture dishes containing increasing DD concentrations. Eggs were treated with three different DD concentrations (0.2, 0.4, 0.6 µg·mL^−1^). After 10 min, eggs were transferred to new 60 mm cell culture dishes containing only filtered seawater and were fertilized. For fertilization, untreated spermatozoa were added at a final concentration of 10^6^ cells to the 60 mm cell culture dishes containing the pretreated eggs. In the second experiment, sperm were treated with three different DD concentrations (0.5, 1.0, 2.0 µg·mL^−1^), which were then used to fertilize virgin untreated oocytes. In this case, spermatozoa were collected directly from the gonoduct, placed in 2 mL Eppendorf conical tubes containing DD solutions, and kept in these solutions for 10 min. An appropriate volume of treated sperm solution was added to egg solutions to fertilize the eggs in 60 mm cell culture dishes. The DD concentrations used to treat sperm were higher than those used to treat eggs and were experimentally selected after preliminary tests. All DD concentrations used in these experiments were experimentally selected through preliminary experiments and the DD concentrations chosen fall within the range normally used in laboratory experiments [17]. In all two treatments, fifteen different experiments were conducted and three replicates for each DD concentrations tested were considered.

### 4.4. Exposure to DD Post-Fertilization

Soon after fertilization, approximately five hundred embryos (for each DD concentrations) were transferred to 60 mm cell culture dishes containing increasing DD concentrations. Another five hundred embryos from the same fertilization event were transferred to other 60 mm cell culture dishes containing only seawater and these were used as a control. In this experiments, five different DD concentrations were tested (0.30, 0.35, 0.40, 0.45, 0.50 µg·mL^−1^). After DD treatment, embryos were incubated to hatching at 21 °C. During development, to evaluate a possible delay in hatching, embryos were observed under the light microscope every thirty minutes up to hatching. Fifteen different experiments were conducted and three replicates for each DD concentrations tested were considered.

### 4.5. Recovery Experiments

Recovery experiments were conducted by collecting eggs from one individual, and fertilizing them with sperm from a different individual. The fertilized eggs were divided in two batches and treated with the same DD concentrations (0.30, 0.50 µg·mL^−1^). All two batches were incubated at 21 °C. The first batch was incubated up to hatching; the second batch was incubated up to the 8–16 cell stage, rinsed twice for five minutes in fresh seawater and incubated at 21 °C up to hatching.

### 4.6. RNA Extraction and cDNA Synthesis

Embryos at the late-tailbud stage (about 9 h post fertilization-hpf) were collected after treatment of newly fertilized eggs with three different DD concentrations 0.35, 0.40, 0.45 µg·mL^−1^; embryos were placed in 500 μL lysis buffer of the RNAqueous-micro kit (Ambion, Grand Island, NY, USA), and frozen at −80 °C until RNA extraction. Embryos were collected from three different experiments (three biological replicates). Total RNA extraction was performed following the manufacturer’s instructions and eluted in 2 × 15 μL of pre-warmed (70 °C) elution buffer. Total RNA (1 μL) was analyzed with the BioAnalyzer (Agilent Technologies, Santa Clara, CA, USA) using the Eukaryote total RNA Micro Series II kit and the mRNA Smear Nano program following the manufacturer’s instructions (Agilent Technologies). Single strand cDNA synthesis from total RNA was obtained by iScript™cDNA Synthesis Kit (Biorad, Hercules, CA, USA). The reaction was carried out following the manufacturer instructions. Briefly, 1 μg of total RNA was resuspended in RNase-free H_2_O in a final volume of 10 μL; this volume was added to 4 μL of iScript reaction mix. Finally, 1 μL of iScript Reverse Transcriptase was added to obtain a final volume of 20 μL. The reaction mix was incubated at different temperatures for different lengths of time as follow: 5' at 25 °C, 30' at 42 °C, 5' at 85 °C.

### 4.7. Quantitative Real-Time PCR (qPCR)

Real time PCR was carried out in order to analyze expression changes between embryos (after treatment on newly fertilized eggs with 0.35, 0.40, 0.45 µg·mL^−1^ DD) *vs.* untreated control embryos. The dye used was Fast Sybr Green Master Mix (Applied Biosystems, Waltham, MA, USA). A pair of primers for the Rps27 ribosomal gene, which is therefore ubiquitary in *Ciona* embryos, was used as a standard reference.

For each gene, qPCR primers were designed to generate products of 100–200 bp, by using online based “Primer 3, v.0.4.0” software (http://fokker.wi.mit.edu/primer3/input.html [27,28]; Table 4). Blast searches against the whole *Ciona* genome were performed to verify primer specificity. The number of cycles needed for the standards to reach a specified Ct was used to normalize the Ct for the selected genes. To capture intra-assay variability all RT-qPCR reactions were carried out in triplicate and the average Ct value was taken into account for further calculations. The efficiency of each pair of primers was calculated according to standard method curves using the equation E = 10^−1/slope^. Five serial dilutions were set up to determine the Ct value and the efficiency of reaction of all pairs of primers. Standard curves were generated for each oligonucleotide pair using the Ct value *vs.* the logarithm of each dilution factor. Diluted cDNA was used as template in a reaction containing a final concentration of 0.70 pmol·μL^−1^ for each primer and 1X Fast SYBR Green master mix (total volume of 10 μL). PCR amplifications were performed in triplicate in a ViiA7 ABI Applied Biosystems thermal cycler, using the following thermal profile: 95 °C for 20'', one cycle for cDNA denaturation; 95 °C for 1'' and 60 °C for 20'', 40 cycles for amplification; 95 °C for 15'', 60 °C for 1' and 95 °C for 15'', one cycle for melting curve analysis, to verify the presence of a single product. Each assay included a no-template control for each primer pair. The relative expression ratio (R) of target genes was calculated based on E (Efficiency) and the Ct deviation of unknown samples *vs.* controls, and expressed in comparison to a reference gene, using the Pfaffl Method [13]. Data for each gene were normalized against Rps27. Changes in gene expression were considered significant only at greater than a 1.5 fold level over controls.

**Table 4:** List of oligonucleotides used for qPCR. The oligo names are indicated in the left column; F letter indicates the Forward oligo, R reverse oligo. The sequence (5'–3') of the oligo primers are indicated in the right column. All qPCR primers (except *cdx*) were designed by using online based “Primer 3, v.0.4.0” software (http://fokker.wi.mit.edu/primer3/input.html). *Cdx* primers were adapted from Hamada *et al.* [29].

Oligo Name	Oligo Sequence
*gclm F*	5'-ATCGTCTCCCTCCCCATATC-3'
*gclm R*	5'-ATCCTTGCCCAATCATTCAA-3'
*gst F*	5'-CAGCGAGAACAGGCTTTACC-3'
*gst R*	5'-AAAAGGTTTCAGCCAGACGA-3'
*hox1F*	5'-CATTGCGCCTTAATGAAACC-3'
*hox1R*	5'-GATGATGACGATGCGAGGTA-3'
*hox12F*	5'-TGGATCATTACGGCTCACAG-3'
*hox12R*	5'-TGGATGATGGTGGTGTGGTA-3'
*cdx F*	5'-AAGGCCGTATGAGTGGATAAG-3'
*cdx R*	5'-TGTCCTTAGTTCGCGTTTTG-3'
*gsx F*	5'-CAAACAGTCATCTCGCCAAG-3'
*gsx R*	5'-GGACGAGCTGTGGAGACTTC-3'

### 4.8. Statistical Analysis

Statistical comparisons between treatments and the corresponding control were carried out using a one-way ANOVA test. A value of *p* < 0.05 was considered statistically significant. To conduct statistical analysis ten different experiments were considered.

## 5. Conclusions

Gene expression represents a unique tool to study how living organisms react and eventually adapt to changes in the habitat where they live. Our results, although preliminary, represent the first attempt to understand the molecular mechanisms of toxicants in a key marine model organism and indicate that the *Ciona* system has the potential to be developed further as a model for molecular developmental toxicity studies. Moreover we are on the way to broaden our horizons by the analysis of DD effects on *Ciona* using a microarray approach, one of the most feasible methods to rapidly inquire into the entire biological response of a whole organism to chemical and drug exposure. The identification of genes with altered expression upon exposure to a chemical can give important clues about the mechanism of action of the toxicant and pave the way for finding general biomarkers to detect exposure to pollutants.
